# Supplementary material for: LcWRKY17, a WRKY Transcription Factor from Litsea cubeba, Effectively Promotes Monoterpene Synthesis
Source: Int J Mol Sci. 2023 Apr 13;24(8):7210. doi: 10.3390/ijms24087210 (PMC10138983; doi:10.3390/ijms24087210)
Supplement: Supplementary file 1 [file ijms-24-07210-s001.zip › ijms-2270933-supplementary.pdf]

Supplementary Data

Table S1. *WRKY* family genes identified in *L. cubeba*.

	Gene ID	Location		chr	clade
LcWRKY1	Lcu01G_02930	141502371	141515878	chr1	Group I
LcWRKY2	Lcu01G_03048	144723698	144733190	chr1	Group I
LcWRKY3	Lcu02G_04012	9342662	9345929	chr2	Group I
LcWRKY4	Lcu02G_04031	9760054	9774247	chr2	Group I
LcWRKY5	Lcu02G_08149	121086513	121103913	chr2	Group I
LcWRKY6	Lcu03G_09263	15956137	15959098	chr3	Group I
LcWRKY7	Lcu04G_12185	71186138	71242706	chr4	Group I
LcWRKY8	Lcu04G_13283	108413185	108416835	chr4	Group I
LcWRKY9	Lcu04G_14344	133267967	133273872	chr4	Group I
LcWRKY10	Lcu04G_14412	134955490	134959901	chr4	Group I
LcWRKY11	Lcu05G_16137	65306507	65321542	chr5	Group I
LcWRKY12	Lcu05G_17192	90588208	90591874	chr5	Group I
LcWRKY13	Lcu05G_18014	105865874	105871244	chr5	Group I
LcWRKY14	Lcu06G_20244	76575186	76597308	chr6	Group I
LcWRKY15	Lcu07G_20738	7183526	7197406	chr7	Group I
LcWRKY16	Lcu07G_22037	71657015	71664500	chr7	Group I
LcWRKY17	Lcu07G_22273	77157816	77187597	chr7	Group I
LcWRKY18	Lcu02G_04000	8816172	8818890	chr2	Group II-a
LcWRKY19	Lcu02G_04001	8831319	8833298	chr2	Group II-a
LcWRKY20	Lcu05G_16732	80868576	80880586	chr5	Group II-a
LcWRKY21	Lcu06G_20389	83138884	83140975	chr6	Group II-a
LcWRKY22	Lcu09G_25580	27313817	27320621	chr9	Group II-a
LcWRKY23	Lcu04G_13446	111864138	111869032	chr4	Group II-b
LcWRKY24	Lcu05G_15061	3933662	3936688	chr5	Group II-b
LcWRKY25	Lcu07G_20654	3134806	3143086	chr7	Group II-b
LcWRKY26	Lcu08G_23653	60015744	60018474	chr8	Group II-b
LcWRKY27	Lcu09G_24736	2508163	2510777	chr9	Group II-b
LcWRKY28	Lcu09G_24740	2564197	2566811	chr9	Group II-b
LcWRKY29	Lcu10G_26091	1235729	1241424	chr10	Group II-b
LcWRKY30	Lcu11G_27988	5300039	5303039	chr11	Group II-b
LcWRKY31	Lcu01G_01130	30991235	31003768	chr1	Group II-c
LcWRKY32	Lcu01G_01177	32860042	32869048	chr1	Group II-c
LcWRKY33	Lcu02G_07106	95872535	95875833	chr2	Group II-c
LcWRKY34	Lcu04G_13655	116234141	116235892	chr4	Group II-c
LcWRKY35	Lcu07G_20574	1003257	1006309	chr7	Group II-c
LcWRKY36	Lcu08G_23890	64896108	64899109	chr8	Group II-c
LcWRKY37	Lcu09G_24792	3356623	3359628	chr9	Group II-c
LcWRKY38	Lcu10G_26179	2962357	2966162	chr10	Group II-c

	Gene ID	Location		chr	clade
LcWRKY39	Lcu10G_26191	3208371	3212186	chr10	Group II-c
LcWRKY40	Lcu10G_27444	45944115	45950900	chr10	Group II-c
LcWRKY41	Lcu02G_04230	14135554	14137531	chr2	Group II-d
LcWRKY42	Lcu02G_04787	27050131	27052585	chr2	Group II-d
LcWRKY43	Lcu03G_10804	123809215	123813376	chr3	Group II-d
LcWRKY44	Lcu03G_11241	152646045	152649740	chr3	Group II-d
LcWRKY45	Lcu04G_13987	126147066	126150855	chr4	Group II-d
LcWRKY46	Lcu05G_16661	78978521	78981452	chr5	Group II-d
LcWRKY47	Lcu05G_16662	78986566	78989000	chr5	Group II-d
LcWRKY48	Lcu06G_18545	5109515	5130714	chr6	Group II-d
LcWRKY49	Lcu08G_24063	68269613	68274060	chr8	Group II-d
LcWRKY50	Lcu01G_01313	42421635	42425485	chr1	Group II-e
LcWRKY51	Lcu02G_06743	89038130	89040899	chr2	Group II-e
LcWRKY52	Lcu04G_11771	16664106	16665742	chr4	Group II-e
LcWRKY53	Lcu05G_18113	107783721	107809316	chr5	Group II-e
LcWRKY54	Lcu07G_21484	59518058	59520501	chr7	Group II-e
LcWRKY55	Lcu08G_23775	62585051	62586448	chr8	Group II-e
LcWRKY56	Lcu09G_24926	6477460	6479246	chr9	Group II-e
LcWRKY57	Lcu10G_26249	4217407	4219243	chr10	Group II-e
LcWRKY58	Lcu04G_11392	5595073	5597421	chr4	Group III
LcWRKY59	Lcu04G_11429	6760389	6762735	chr4	Group III
LcWRKY60	Lcu04G_11430	6781827	6782749	chr4	Group III
LcWRKY61	Lcu04G_11887	21700657	21703134	chr4	Group III
LcWRKY62	Lcu04G_13190	106090776	106094773	chr4	Group III
LcWRKY63	Lcu04G_13191	106110797	106113089	chr4	Group III
LcWRKY64	Lcu10G_27409	43837240	43840528	chr10	Group III

Table S2. Regulation of terpenoid biosynthesis involving WRKY transcription factors

Species	WRKY	Function	References
Malus domestica	*MdWRKY40*	anthocyanin biosynthesis	23
Artemisia annua	*AaWRKY1*	artemisinin biosynthesis	24
Arabidopsis thaliana	*AtWRKY41*	anthocyanin biosynthesis	25
Arabidopsis thaliana	*AtWRKY44*	*secondary metabolite synthesis*	*26*
Gossypium arboretum	*GaWRKY1*	terpene biosynthesis	27
Nicotiana tabacum	*NaWRKY3/NaWRKY6*	terpene biosynthesis	30
Solanum lycopersicum	*SIWRKY71*	terpene biosynthesis	60
Solanum lycopersicum	*SIWRKY35*	monoterpene biosynthesis	36

Table S3. Sequences of primers

	Primer Names	Primer Sequence（5'-3'）
LcWRKY17 gene cloning	LcWRKY17-PF	GATATGCGTGCACCGATTAACG
	LcWRKY17-PR	AACGCCTTAGATCTTTTCATGACAT
LcWRKY17 vector construction	NC- LcWRKY17-PF	agtggtctctgtccagtcctGATATGCGTGCACCGATTAA
	NC- LcWRKY17-PR	ggtctcagcagaccacaagtAACGCCTTAGATCTTTTCAT
qRT-PCR verification	LcWRKY17-qRT-F	TCAAGGGCAACTGCTTCTTT
	LcWRKY17-qRT-R	GTCATATGGGTTTGGGGATG
	LcTPS42-qRT-F	GTTGTCCTCAGCGGCTTCTT
	LcTPS42-qRT-R	GCTTGGATCGAATGGAGCAT
	UBC-F	TGTGTGTGTGTGTGTGTGTCC
	UBC-R	CCTTTCTCCACGGTCTTCAA

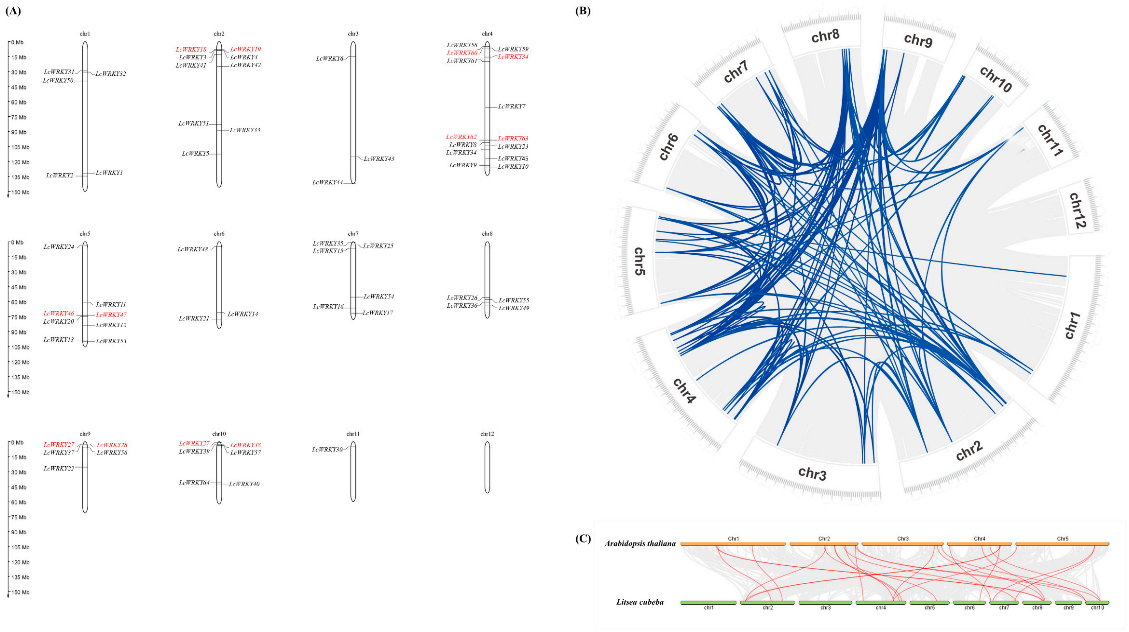

Figure S1. Chromosomal distribution and gene duplication events of *LcWRKYs*. (A) The position of *LcWRKYs* on the chromosome. Scale bar on the left indicates length of *L. cubeba* chromosome (Mb). Red font represents tandem repeat genes. (B) Genome wide synteny analysis of *LcWRKYs*. Segmental duplication events are linked by blue lines. (C) Genome wide synteny analysis of *AtWRKYs* and *LcWRKYs*. Segmental duplication events are linked by red lines.

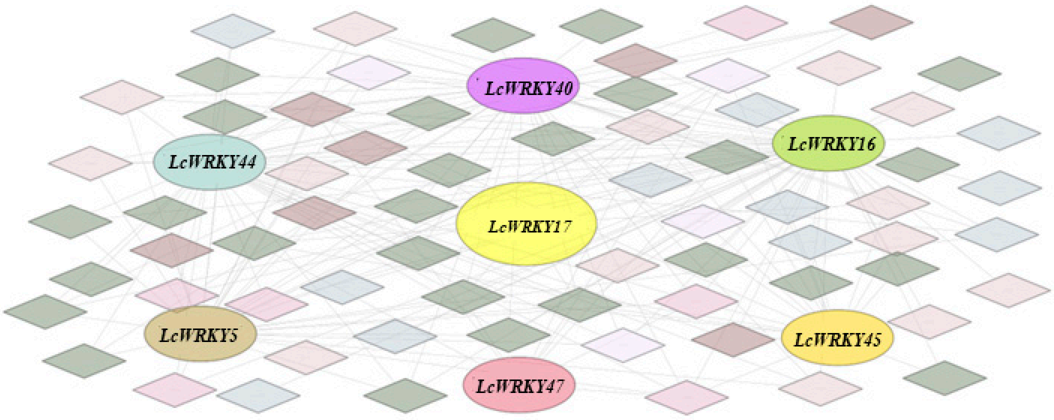

Figure S2. *LcWRKYs* gene expression network. The different colors in the diamond represent different secondary metabolic pathways, including terpenoid backbone biosynthesis, monoterpenoid biosynthesis, sesquiterpenoid and triterpenoid biosynthesis, indole alkaloid biosynthesis, brassinosteroid biosynthesis and metabolic pathways.
